# Prevalence and correlates of active syphilis diagnosed using serological assays and the molecular research-use-only Aptima *Treponema pallidum* assay among pregnant women in Zambia, 2023

**DOI:** 10.3389/fpubh.2026.1758573

**Published:** 2026-04-14

**Authors:** Elena Shipitsyna, Sumire Sorano, Daniel Schröder, Enesia Banda Chaponda, Daniel Golparian, Ephraim Chikwanda, Vivian Mwewa, Joyce M. Mulenga, Mike Chaponda, Chris Smith, Mitsuaki Matsui, Massimo Mirandola, Karel Blondeel, Igor Toskin, R. Matthew Chico, Magnus Unemo

**Affiliations:** 1 WHO Collaborating Centre for Gonorrhoea and Other STIs, Department of Laboratory Medicine, Faculty of Medicine and Health, Örebro University, Örebro, Sweden; 2 Department of Medical Microbiology, D. O. Ott Research Institute of Obstetrics, Gynecology and Reproductive Medicine, St. Petersburg, Russia; 3 Faculty of Infectious and Tropical Diseases, London School of Hygiene and Tropical Medicine, London, United Kingdom; 4 School of Tropical Medicine and Global Health, Nagasaki University, Nagasaki, Japan; 5 Department of Biosciences and Biotechnology, School of Natural and Applied Sciences, University of Zambia, Lusaka, Zambia; 6 National Health Research and Training Institute, Ndola, Zambia; 7 St. Paul's Mission Hospital, Nchelenge, Zambia; 8 Division of Global Health, Department of Public Health, Graduate School of Health Sciences, Kobe University, Kobe, Japan; 9 Department of Diagnostics and Public Health, University of Verona, Verona, Italy; 10 Faculty of Medicine and Health Sciences, Ghent University, Ghent, Belgium; 11 Department of Sexual and Reproductive Health and Research, World Health Organization, Geneva, Switzerland; 12 WHO Collaborating Centre for Sexually Transmitted Infections, London School of Hygiene and Tropical Medicine, London, United Kingdom; 13 Institute for Global Health, University College London (UCL), London, United Kingdom

**Keywords:** antenatal population, epidemiologic correlates, prevalence, syphilis, transcription-mediated amplification, *Treponema pallidum*, treponemal and non-treponemal tests, Zambia

## Abstract

**Introduction:**

Syphilis is a leading cause of adverse pregnancy outcomes, excessively affecting sub-Saharan Africa. Early diagnosis and treatment are crucial to prevent mother-to-child transmission. The aims of the present study were to assess prevalence and epidemiological and clinical correlates of active syphilis diagnosed using serological [treponemal and non-treponemal (lipoidal)] assays and the research-use-only (RUO) Aptima *Treponema pallidum* assay [Hologic; transcription-mediated amplification (TMA) assay] among pregnant women attending four antenatal care facilities in Nchelenge, Zambia in 2023.

**Methods:**

Syphilis serology was performed using rapid diagnostic test [RDT; Core tests^®^ ONE STEP Syphilis Test Kit (Core Technology, Atlanta, USA)]; positive samples were further evaluated by rapid plasma reagin [RPR; RPR Carbon Antigen Reagent (Eurocarb Products Ltd., Bristol, United Kingdom)] test. Active syphilis was defined as a positive RDT plus a positive RPR test, or a positive RUO Aptima *T. pallidum* assay. *T. pallidum* and other non-viral STIs (*Chlamydia trachomatis, Neisseria gonorrhoeae, Mycoplasma genitalium*, and *Trichomonas vaginalis*) were detected in clinician-collected vaginal swab samples using Aptima assays on the Panther system (Hologic). HIV testing was performed with the Determine HIV Test Kit, and bacterial vaginosis (BV) was diagnosed using Nugent's score. Active syphilis correlates were identified using univariable and multivariable logistic regression.

**Results:**

In total, 996 pregnant women were included in the analysis, of whom 136 women (13.7%) had an active syphilis infection. Of these women with active syphilis, 117 (86.0%) were positive in serology only, 15 (11.0%) in serology plus RUO Aptima *T. pallidum* assay, and four (2.9%) in RUO Aptima *T. pallidum* assay only. Rates of other current STIs and reproductive tract infections included: BV (23.3%), *T. vaginalis* (22.7%), *M. genitalium* (12.7%), HIV (8.7%), *N. gonorrhoeae* (8.4%), and *C. trachomatis* (7.4%). Secundigravidity, history of stillbirth, current concomitant *M. genitalium, N. gonorrhoeae, T. vaginalis*, and HIV infections were significant risk factors of active syphilis. Advanced gestational age (from 28 to 39 weeks) at enrolment was associated with significantly lower risk of syphilis positivity.

**Discussion:**

A remarkably high burden of syphilis and high co-infection rates with HIV and other non-viral STIs in pregnant women in Zambia underscore the urgent need for integrating syphilis and HIV universal screening programs, as well as to consider concurrent testing for selected other non-viral STIs. Where feasible due to the increased cost, supplementing serological screening with a highly sensitive and specific molecular test such as the RUO Aptima *T. pallidum* assay for suspected early syphilis can ensure timely detection of both seroconverted and seronegative early syphilis cases, early treatment and effective prevention of mother-to-child transmission.

## Introduction

Active maternal syphilis substantially increases the risk of serious obstetric and neonatal complications including stillbirth, miscarriage, preterm delivery, low birthweight, and congenital malformations (1). Timely diagnosis and treatment of all infected pregnant women can prevent nearly all serious adverse outcomes of congenital syphilis and is a standard of care recommended by national and global public health authorities (2, 3). To achieve significant reductions in the global burden of syphilis infection in pregnancy as part of the World Health Organization (WHO) Triple Elimination Initiative (4, 5), efforts must be redoubled in sub-Saharan Africa (SSA) where syphilis continues to impose a severe and disproportionately high burden of disease. A 2019 meta-analysis showed a pooled prevalence of 2.9% in antenatal care (ANC) settings across SSA (6). In urban Lusaka, Zambia, studies showed a prevalence of maternal syphilis ranging from 2.2% (7) to 4.8% (8). In rural Nchelenge, however, the prevalence has been shown to be considerably higher at 7.1% (9).

The WHO guideline for syphilis in pregnancy suggests that in resource-limited settings, where: (i) ANC screening coverage is low, (ii) follow-up is challenging, and/or (iii) laboratory capacity is limited, on-site rapid diagnostic tests (RDTs) should be deployed (2). The guideline recommends using a treponemal RDT and, if RDT-positive, administering an initial benzathine penicillin G (BPG) treatment dose immediately to prevent adverse pregnancy outcomes. In high-prevalence settings (≥5%), women who are RDT-positive for syphilis should be subsequently tested with a quantitative rapid plasma reagin (RPR) test to guide further treatment (2). Notably, in Zambia screening using a syphilis RDT is recommended to be performed at the first ANC visit, which is in accordance with WHO guidelines (2). Despite these recommendations for universal screening and same-day treatment by WHO and in national guidelines, syphilis screening rates in ANC settings in Zambia remain low, ranging between 28% and 43% in recent studies (7, 8). Barriers to syphilis diagnosis and prompt treatment in ANC settings include frequent stockouts of syphilis test kits and BPG at the facility level, which critically undermines efficacy of detection and treatment of syphilis, and prevention of mother-to-child transmission (7, 8). In a recent cohort study among women diagnosed with syphilis in health facilities in Zambia, only 48% had BPG treatment documented by clinical records (10).

The natural course of syphilis includes primary, secondary, and tertiary stages, additionally there are early and latent stages. Primary, secondary and early latent infections are considered as sexually transmissible stages of syphilis, which occur during the first 2 years after infection and is referred to as early (active) syphilis. The primary and secondary stages present the greatest risk for mother-to-child transmission, while the early latent stage is associated with a lower risk (11).

The diagnosis of syphilis is challenging because of its multi-stage clinical course and the limitations of available testing methods. Its presentation is characterized by highly variable, transient symptoms followed by extended latent periods, often complicating clinical diagnosis due to symptoms overlapping with other conditions (12). Consequently, for diagnosis of syphilis laboratory or RDT testing is required, and serology is the primary tool providing a presumptive diagnosis. Treponemal serological tests, like *Treponema pallidum* particle agglutination (TP-PA), enzyme immunoassay (EIA), and most treponemal RDTs, typically remain positive for life, even after successful treatment. Non-treponemal (lipoidal) tests, such as RPR or venereal disease research laboratory (VDRL) tests, are markers for disease activity and treatment response, as their titers decrease substantially post-treatment (13). In active infections, both treponemal and non-treponemal tests are typically reactive. For direct detection of *T. pallidum* providing a definitive syphilis diagnosis in early symptomatic stages, nucleic acid amplification tests (NAATs) are now preferred over dark-field microscopy due to their superior sensitivity (14–16). In early infection, NAATs for *T. pallidum* can be positive before seroconversion, which typically happens within 3 weeks, but can take up to 90 days (15). A number of PCR assays have been developed, and a recent systematic review and meta-analysis estimated that 10% (range 4% to 20%) of primary syphilis cases would have been missed if serology alone was used (16). A novel research-use-only (RUO) NAAT has recently been developed for detection of *T. pallidum* 23S rRNA using real-time transcription-mediated amplification (TMA) (RUO Aptima *T. pallidum* assay; Hologic, San Diego, CA, USA), which has shown high analytical and diagnostic performance (17–19).

The aims of the present study were to assess the prevalence and epidemiological and clinical correlates of active syphilis diagnosed using serological (treponemal and non-treponemal) assays and the RUO Aptima *T. pallidum* assay among pregnant women attending four ANC facilities in Nchelenge, Zambia in 2023.

## Materials and methods

### Study population and specimen collection

This was a nested study within a registered clinical trial (PACTR202302766902029) evaluating point-of-care RDTs for *T. vaginalis* and bacterial vaginosis (BV). It was conducted between February 15 and May 26, 2023, at four ANC facilities in Nchelenge, Zambia. Pregnant women were recruited if they: (i) were at least 13 weeks of gestation; (ii) had no self-reported use of metronidazole or clindamycin during the current pregnancy; (iii) had no known allergies or contraindications to metronidazole; and (iv) reported no use of vaginal creams or ointments, douching, or vaginal lubricants within 72 h prior to recruitment. All women provided written informed consent before sample collection and the completion of a clinical background questionnaire. From each woman, capillary blood was obtained by finger prick for RDTs and vaginal swabs were collected by clinical staff using the Aptima Vaginal Swab Specimen Collection kit (Hologic). The baseline characteristics, relevant for the present study, reported by participating women through the clinical background questionnaire included age, ANC facility, relational status, gestational age, number of previous pregnancies, history of preterm birth, history of miscarriage, and history of stillbirth. Results of the parent study have been published elsewhere (20).

### Testing for syphilis and other STIs

Serological testing for syphilis and HIV was performed as part of routine ANC. The RDT Core tests^®^ ONE STEP Syphilis Test Kit (Core Technology, Atlanta, USA) was used for syphilis screening, and all samples positive with the RDT were tested using a qualitative RPR test (RPR Carbon Antigen Reagent; Eurocarb Products Ltd., Bristol, United Kingdom). A sample positive with the RDT plus RPR test, as well as samples positive in the RUO Aptima *T. pallidum* assay, irrespective of serology results, were interpreted as representing active syphilis. Positive RDT followed by negative RPR test was considered a previously treated or latent syphilis case. HIV was detected using Determine HIV-1/2 Test Kit (Abbott Laboratories, Chicago, IL, USA).

For the detection of *T. pallidum, Chlamydia trachomatis, Neisseria gonorrhoeae, Mycoplasma genitalium*, and *T. vaginalis* in the vaginal swab samples, the RUO Aptima *Treponema pallidum* assay, Aptima Combo 2 assay, Aptima *Mycoplasma genitalium* assay, and Aptima *Trichomonas vaginalis* assay (Hologic) on the Panther System (Hologic) were used, respectively, in accordance with the manufacturer's instructions. BV was diagnosed using Nugent's scores.

### Statistics

All numerical variables were categorized as having non-normal distribution using the Shapiro–Wilk test and presented by medians with interquartile range and overall range. Categorical variables were presented by percentages with 95% confidence intervals (95% CIs). For measuring the degree of association of selected background and clinical variables with active syphilis, crude odds ratios (cORs) were computed using univariable logistic regression. A multivariable logistic regression analysis included variables with a significance level of *P* ≤ 0.200 in the univariable analysis.

Statistical analyses were performed with the use of the statistics package IBM SPSS Statistics 27 (IBM). All tests for significance were two-sided, and statistically significant differences were assumed when *P* < 0.05.

## Results

A total of 1,021 women were enrolled in the parent study (20). Briefly, 149 (14.7%) of 1,017 available blood samples tested positive using the syphilis RDT (blood samples from four women were unavailable) (Tables 1, 2). Eighteen of these 149 RDT-positive blood samples were unavailable for subsequent testing with the RPR test; 129 (98.5%) of the 131 available RDT-positive samples were positive also in the RPR test. In the RUO Aptima *T. pallidum* assay, 19 (1.9%) of 1,017 available vaginal swab samples were positive for *T. pallidum* (vaginal swab samples from four women were unavailable) (Tables 1, 2). Of these 19 RUO Aptima *T. pallidum* assay-positive samples, 15 samples were from RDT-positive women and four samples were from RDT-negative women. In total, 25 women were excluded from subsequent statistical analysis because they were interpreted as having previously treated syphilis (*n* = 2) or unknown syphilis status (*n* = 23). Consequently, 996 women were included in the analysis: 136 (13.7%) women interpreted as having active syphilis (RDT plus RPR test positive, and/or RUO Aptima *T. pallidum* assay positive) and 860 (86.3%) women negative for syphilis. Of the women with active syphilis, 117 (86.0%) were positive in serology only, 15 (11.0%) in serology plus RUO Aptima *T. pallidum* assay, and four (2.9%) in RUO Aptima *T. pallidum* assay only. Table 2 summarizes the detection rates with the different syphilis tests: 14.7% of women were positive with the RDT, 13.7% with the RDT plus RPR test, 1.5% with the RDT plus RUO Aptima *T. pallidum* assay, and 0.4% with the RUO Aptima *T. pallidum* assay only (incubating syphilis).

**Table 1:** Results of syphilis tests (rapid diagnostic test, rapid plasma reagin test, and research-use-only Aptima *Treponema pallidum* assay) in women attending antenatal care in Zambia (*n* = 1,021).

RDT	RPR	RUO Aptima *T. pallidum* assay	Interpretation	No. of samples
Positive	Positive	Positive	Active	12
Positive	Positive	Negative	Active	117
Positive	Unavailable^a^	Positive	Active	3
Negative	NA^b^	Positive	Active	4
Total with active syphilis				136
Negative	NA^b^	Negative	Negative	860
Total syphilis negative				860
Positive	Negative	Negative	Treated or latent^c^	2
Positive	Unavailable^a^	Negative	Unknown^c^	15
Negative	NA^b^	Unavailable^a^	Unknown^c^	4
Unavailable^a^	Unavailable^a^	Negative	Unknown^c^	4
Total excluded samples				25

RDT, rapid diagnostic test; RPR, rapid plasma reagin; RUO, research-use-only; No., number; NA, not applicable.

^a^Sample or testing result unavailable.

^b^Not applicable because RDT negative.

^c^Excluded from statistical association analysis.

**Table 2:** Syphilis positivity with rapid diagnostic test, rapid plasma reagin test, and research-use-only Aptima *Treponema pallidum* assay in women attending antenatal care in Zambia.

Test(s)	No. positive/No. tested	% (95% CI)
RDT positive	149/1,017	14.7 (12.6–17.0)
RUO Aptima *T. pallidum* assay positive	19/1,017	1.9 (1.2–2.9)
RDT positive and RPR positive, or RUO Aptima *T. pallidum* assay positive (active syphilis)	136/996	13.7 (11.7–15.9)
RUO Aptima *T. pallidum* assay positive and RDT negative (incubating syphilis)	4/996	0.4 (0.2–1.0)

CI, confidence interval; RDT, rapid diagnostic test; RPR, rapid plasma reagin; RUO, research-use-only.

Baseline characteristics of the study participants are summarized in Table 3. Fifty-eight percent of the women were younger than 25 years. The majority of the women (71%) were married or living with a partner, and 30% were primigravida. Fifty-seven percent of the women were enrolled between 13 and 27 weeks of gestation, and 43% between 28 and 39 weeks. History of preterm birth, miscarriage, and stillbirth was reported by 1.6%, 6.2%, and 4.2% of the women, respectively.

**Table 3:** Baseline characteristics of women included in statistical association analysis (*n* = 996).

Characteristics	Median (IQR; range) or percentage (95% CI), *n*/*n* total
Age, years	23 (20–30, 14–46)
Age in category, years	
< 25	57.7 (54.7–60.8), 575/996
≥25	42.3 (39.2–45.4), 421/996
Relational status	
Married/partnered	71.2 (68.3–73.9), 708/995
Single/separated/divorced/widowed	28.8 (26.1–31.7), 287/995
Gestational age at enrolment, weeks	26 (21–32; 13–39)
Gestational age at enrolment in category	
13–27 week	57.0 (53.9–60.1), 568/996
28–39 week	43.0 (39.9–46.1), 428/996
No. of previous pregnancies	2 (1–4; 1–9)
No. of previous pregnancies in category	
0	30.3 (28.2–35.1), 302/996
1	22.0 (27.69–33.3), 219/996
2	17.4 (15.1–19.9), 173/996
3	14.8 (12.7–17.1), 147/996
≥4	15.6 (13.4–17.9), 155/996
History of preterm birth^a^	1.6 (0.9–2.8), 11/693
History of miscarriage^a^	6.2 (4.6–8.3), 43/693
History of stillbirth^a^	4.2 (2.9–5.9), 29/694

IQR, interquartile range; CI, confidence interval.

^a^Computed for multigravida women.

Apart from syphilis, the rates of other current STIs and reproductive tract infections were: BV (23.3%), *T. vaginalis* (22.7%), *M. genitalium* (12.7%), HIV (8.7%), *N. gonorrhoeae* (8.4%), and *C. trachomatis* (7.4%) (Table 4).

**Table 4:** Prevalence of sexually transmitted infections in women attending antenatal care in Zambia.

Sexually transmitted infection	Percentage (95% CI), *n*/*n* total
*Chlamydia trachomatis*	7.4 (5.9–9.2), 73/990
*Neisseria gonorrhoeae*	8.4 (6.8–8.3), 83/991
*Mycoplasma genitalium*	12.7 (10.7–14.9), 124/980
*Trichomonas vaginalis*	22.7 (20.2–25.5), 226/994
Bacterial vaginosis	23.3 (20.8–26.1), 228/977
HIV	8.7 (7.1–10.6), 86/987
Syphilis	13.7 (11.7–15.9), 136/996

CI, confidence interval.

In univariable logistic regression, being single, or separated or divorced, or widowed (compared to married or partnered), multiple previous pregnancies (1, 2, or 3 compared to 0), history of stillbirth, current concomitant *M. genitalium, N. gonorrhoeae, T. vaginalis*, and HIV infections were significant risk factors of active syphilis, whereas advanced gestational age at enrolment (from 28 to 39 weeks compared to 13 to 27 weeks) was a significant protective factor (Table 5). In multivariable logistic regression model, being secundigravida, having a history of stillbirth, and current *M. genitalium, N. gonorrhoeae, T. vaginalis*, and HIV infections were independent risk factors for active syphilis, and being 25 years of age or older at enrolment remained significant protective factor (Table 5).

**Table 5:** Baseline characteristics associated with active syphilis in women attending antenatal care in Zambia (*N* = 996).

Factors and categories	Syphilis positivity % (95% CI), *n*/*n* total	cOR (95% CI)	*P*-value	aOR (95% CI)	*P*-value
Age, years					
< 25	14.6 (12.0–17.7), 84/575	1			
>25	12.4 (9.5–15.8), 52/421	0.824 (0.568–1.194)	0.306		
Relational status					
Married/partnered	15.4 (12.9–18.2), 109/708	1			
Single/separated/divorced/widowed	9.4 (6.6–13.3), 27/287	0.571 (0.365–0.891)	**0.014**	0.800 (0.430–1.489)	0.482
Gestational age at enrolment					
13–27 week	15.7 (12.9–18.9), 89/568	1		1	
28–39 week	11.0 (8.4–14.3), 47/428	0.664 (0.455–0.969)	**0.034**	0.631 (0.426–0.937)	**0.022**
No. of previous pregnancies					
0	7.6 (5.1–11.2), 23/302	1			
1	21.0 (16.1–26.9), 46/219	3.225 (1.889–5.509)	< 0.001	3.497 (1.768–6.917)	**< 0.001**
2	15.6 (11.0–21.8), 27/173	1.992 (1.211–3.276)	**0.007**	2.091 (0.982–4.456)	0.056
3	13.6 (9.0–20.1), 20/147	1.910 (1.012–3.604)	**0.046**	1.974 (0.880–4.427)	0.099
≥4	12.9 (8.5–19.1), 20/155	1.797 (0.954–3.386)	0.070	1.732 (0.770–3.897)	0.184
History of preterm birth^a^					
No	16.4 (13.8–19.4), 112/682	1			
Yes	9.1 (1.6–37.7), 1/11	0.509 (0.065–4.015)	0.522		
History of miscarriage^a^					
No	15.7 (13.1–18.7), 102/650	1		1	
Yes	25.6 (14.9–40.2), 11/43	1.847 (0.902–3.782)	0.094	1.931 (0.953–3.915)	0.068
History of stillbirth^a^					
No	15.5 (12.9–18.4), 103/665	1		1	
Yes	34.5 (19.9–52.7), 10/29	2.872 (1.298–6.353)	**0.009**	2.485 (1.068–5.780)	**0.035**
*Chlamydia trachomatis*					
Negative	13.5 (11.5–15.9), 124/917	1			
Positive	16.4 (9.7–26.6), 12/73	1.258 (0.659–2.403)	0.487		
*Neisseria gonorrhoeae*					
Negative	12.8 (10.8–15.1), 116/908	1		1	
Positive	24.1 (16.2–34.3), 20/83	2.167 (1.264–3.717)	**0.005**	1.977 (1.093–3.575)	**0.024**
*Mycoplasma genitalium*					
Negative	12.4 (10.3–14.8), 106/856	1		1	
Positive	23.4 (16.8–31.6), 29/124	2.160 (1.360–3.431)	**0.001**	1.793 (1.094–3.575)	**0.021**
*Trichomonas vaginalis*					
Negative	12.1 (10.0–14.6), 93/768	1		1	
Positive	19.0 (14.5–24.7), 43/226	1.705 (1.147–2.536)	**0.008**	1.761 (1.144–2.712)	**0.010**
Bacterial vaginosis					
Negative	14.0 (11.7–16.7), 105/749	1			
Positive	13.6 (9.8–18.7), 31/228	0.965 (0.627–1.486)	0.872		
HIV					
Negative	12.4 (10.4–14.8), 112/901	1		1	
Positive	26.7 (18.5–36.9), 23/86	2.572 (1.534–4.312)	**< 0.001**	2.496 (1.420–4.387)	**0.001**

CI, confidence interval; cOR, crude odds ratio; aOR, adjusted odds ratio. Significant *p*-values are in bold letters.

^a^adjusted for relational status, gestational age at enrolment, *Mycoplasma genitalium, Neisseria gonorrhoeae, Trichomonas vaginalis*, HIV.

## Discussion

The present study found a remarkably high prevalence of active syphilis, 13.7% (136/996), among women attending ANC facilities in Zambia. Genetic testing using the RUO Aptima *T. pallidum* assay (Hologic) revealed four women with seronegative syphilis, constituting 2.9% (4/136) of all active syphilis cases diagnosed. Secundigravidity, history of stillbirth, current concomitant *M. genitalium, N. gonorrhoeae, T. vaginalis*, or HIV infections were independent risk factors for active syphilis. Advanced gestational age at enrolment was independently associated with significantly lower risk of active syphilis.

Pregnant women with active syphilis were at a significantly higher risk of being co-infected with HIV compared to women without syphilis. This finding is consistent with the well- documented biological and epidemiological association between syphilis and HIV co-infection (21). A recent systematic review and meta-analysis reported a high syphilis rate (7.1%) among pregnant women living with HIV in SSA (22). However, a substantial disparity exists in the coverage of HIV and syphilis screening programmes in ANC facilities. HIV testing is relatively high [63%−74% in SSA, and 88% in Zambia (23, 24)], yet syphilis screening is much less prioritized [28%−43% coverage in Zambia (7, 8)]. This creates a critical missed opportunity for prevention of syphilis and its transmission to children. Therefore, the integration of HIV and syphilis universal screening services is widely advocated as a necessary strategy to harmonize care, improve resource efficiency, and ensure comprehensive maternal and child health protection (4, 5, 25, 26).

The present study also revealed a high burden of *T. vaginalis* (22.7%), *M. genitalium* (12.7%), and *N. gonorrhoeae* (8.4%) in this ANC population in Zambia, which is largely consistent with a recent meta-analysis among pregnant women in SSA (27). Furthermore, these infections were strongly associated with active syphilis in our study. Due to this high co-infection rate, syphilis diagnosis may necessitate for considering to screen for also additional selected STIs, and vice versa. Unlike the other STIs in our study, *C. trachomatis* was not associated with syphilis. Association between syphilis and several additional STIs is well-established, which is mainly due to common risks and behaviors as well as underlying biological factors; however, the degree of this association is highly variable. For instance, a recent large retrospective cohort of reproductive-aged women demonstrated significant differences in the cumulative incidence of syphilis following another STI diagnosis within a 5-year period: syphilis was diagnosed in approximately 1 of 150 women with chlamydia, 1 of 100 with gonorrhea, and 1 of 50 with HIV (28).

Prevalence of active syphilis in the present study differed significantly by gravidity: the rate was 7.6% in primigravidae and was highest among secundigravidae at 21.0%, decreasing gradually with subsequent pregnancies. Association of syphilis with the number of previous pregnancies has been investigated in several previous studies, but the results have been inconsistent. Some studies showed a higher prevalence in multigravidae (29), while others found no significant difference (30, 31). A higher number of previous pregnancies do not appear to be a direct risk factor for syphilis. Instead, it can be a proxy for cumulative exposure to risk factors and potential gaps in ANC access. This often leads to missed opportunities for screening and treatment, thereby increasing the risk of adverse birth outcomes.

Syphilis is a leading cause of adverse pregnancy outcomes, with pooled estimates of stillbirth and miscarriage in women with untreated syphilis of 26.4% and 14.9%, respectively (32). Our findings comport with this, showing a high rate of active syphilis in women with a history of stillbirth and miscarriage (34.5% and 25.6%, respectively). Stillbirth in previous pregnancies was shown to be a strong independent risk factor of active syphilis, whereas the association with a history of miscarriage did not reach statistical significance.

In contrast to the expectation that syphilis would be more prevalent among women who enrolled later in pregnancy due to ongoing risk in settings with suboptimal care (31), we found the risk of having active syphilis to be lower among women enrolled in the third trimester compared to second-trimester enrollees. We hypothesize that this may be because undiagnosed syphilis leads to pregnancy loss before the third trimester. Supporting this, our data show that one in three women with a prior stillbirth and one in four with a prior miscarriage had active syphilis.

Previous research into the association between relational status and the risk of syphilis indicates that marriage can be a protective factor, largely by promoting monogamous relationships (31, 33). However, in this setting our analysis found no significant protective effect against active syphilis associated with being married or partnered. Moreover, the risk of syphilis was significantly lower in single, separated or divorced, or widowed, as compared to married or partnered women, although in the adjusted regression significance was not reached. Relational status itself may not necessarily be protective against active syphilis, whereas factors such as duration of marriage, age disparities, difference in education level, and condom use can be determinants of syphilis infection in both men and women (34).

Unlike other non-viral STIs, the diagnosis of syphilis relies primarily on serology. Direct diagnostic methods, such as NAATs, are mainly employed when serological results are inconclusive or difficult to interpret, particularly in early syphilis during the “seronegative window” where a chancre is present but antibodies are undetectable (14). Direct detection of *T. pallidum* from ulcers now routinely relies on highly sensitive and specific NAATs, rather than on microscopy (14). Our study underscores the utility of this approach: RUO Aptima *T. pallidum* assay testing even from vaginal swab samples identified active syphilis in four seronegative women, accounting for 2.9% (4/136) of all women diagnosed with active syphilis in the present study. These syphilis cases would have been missed if serology only had been used.

NAATs offer high sensitivity and specificity, making them particularly valuable for diagnosing primary syphilis before seroconversion, as well as for detecting congenital syphilis in neonates, where maternal antibodies can confound serological results. In primary syphilis, the sensitivity and specificity of routine PCR tests range from 78.4% to 89.1% and 93.1% to 100%, respectively (35). NAATs are significantly more sensitive than direct detection methods like microscopy. For instance, a recent study of suspected syphilis lesions compared PCR with immunofluorescence and dark-field microscopy on 809 specimens. The PCR demonstrated a 99.5% sensitivity, vastly outperforming immunofluorescence at 52.4% and dark-field microscopy at 52.9% (36).

The performance of NAATs is highly dependent on the specimen type. Acute mucocutaneous lesions from primary syphilis are the preferred sample, with a reported sensitivity of 95.2%. However, sensitivity decreases to 69.4% in secondary syphilis (37). Recent advancements in syphilis NAATs include the development of the RUO Aptima *T. pallidum* assay targeting the 23S rRNA of *T. pallidum*, which has demonstrated high analytical sensitivity and specificity (17). In a study involving men who have sex with men, the integration of the RUO Aptima *T. pallidum* assay into routine diagnostics identified 14 additional syphilis infections out of 191 patients (7.3% more), highlighting its potential to enhance case finding (18). Our results elucidate the utility of the RUO Aptima *T. pallidum* assay for the diagnosis of active syphilis in an ANC population. The RUO Aptima *T. pallidum* assay testing identified four seronegative women who were confirmed to have syphilis, accounting for 2.9% (4/136) of cases that would have been missed by serology alone.

Key strengths of the present study included the large sample size and the use of a comprehensive algorithm for syphilis diagnostics, which included screening samples with a treponemal test (RDT), further testing of RDT-positive samples with a non-treponemal test (RPR), and additionally using a direct, highly sensitive and specific syphilis test (RUO Aptima *T. pallidum* assay). The limitations and potential sources of biases included the cross-sectional design, which precluded monitoring of treatment efficacy through RPR test titres for any women that had received treatment, the use of only qualitative RPR test, the unavailability of some samples for testing with all diagnostic assays, that the sample size calculation and subsequent sample collection were performed for the registered clinical trial evaluating point-of-care RDTs for *T. vaginalis* and BV (PACTR202302766902029; 20), the lack of standardized comprehensive management of all the RDT-positive women (treatment was provided the same day based on the results of the RDT; but contact tracing, behavioral or preventive counseling or distribution of condoms were not systematically performed for all women, despite that all management was performed in accordance with the standard clinical protocols at the Health Centers), and that the exact number of ANC visits for each participating woman was unknown.

In summary, this study revealed a remarkably high burden of syphilis, as well as high co-infection rates with HIV and other STIs, in the ANC population in Zambia, underscoring the urgent need for integrating syphilis and HIV universal screening programs, as well as to perform concurrent testing for additional selected STIs. Where feasible due to the increased cost, supplementing serological screening for syphilis with a highly sensitive and specific molecular test such as the RUO Aptima *T. pallidum* assay for suspected early syphilis can ensure timely detection of both seroconverted and seronegative early syphilis cases, early treatment and effective prevention of mother-to-child transmission.

## Data Availability

The original contributions presented in the study are included in the article/supplementary material, further inquiries can be directed to the corresponding author.
